# Changing Medical Student Perceptions of Mental Illness Through a Psychiatry Clinical Clerkship: A Longitudinal Qualitative Study

**DOI:** 10.1007/s40596-024-02035-0

**Published:** 2024-09-09

**Authors:** Amy E. Manley, Lucy Biddle, Jelena Savović, Paul Moran

**Affiliations:** 1 Population Health Sciences, Bristol Medical School, University of Bristol, Bristol, UK; 2 NIHR Applied Research Collaboration West (ARC West) at University Hospitals Bristol and Weston NHS Foundation Trust, Bristol, UK

**Keywords:** Medical student, Attitudes, Stigma, Mental illness, Psychiatry

## Abstract

**Objective:**

This study sought to understand whether perceptions of mental illness change during the course of students’ psychiatry clerkships, and what facilitates such change.

**Methods:**

Using a longitudinal qualitative study design, the authors followed up 14 medical students, interviewing them before, during, and after their psychiatric clerkship.

**Results:**

Prior to clerkships, students perceived psychiatric patients to be dangerous, fragile, hard to treat, and to exert a disproportionate emotional toll on clinicians. Stigma was reinforced by safety measures including the provision of alarms, but this improved following “real life” engagement with patients. Students experienced little emotional distress from clinical contacts, particularly those where they led the consultation. Pre-existing beliefs about mental illness being hard to “fix” showed less change over time. Although uncommon, when staff referred to patients using pejorative language, students emulated these negative attitudes.

**Conclusions:**

Among medical students, direct patient contact plays an important role in counteracting pre-existing negative attitudes towards mental illness. This can be facilitated by supportive supervisors, clinical teams allocating students a clear practical role, involving patients in teaching, and roleplay to alleviate potential concerns about seeing patients.

People experiencing serious mental illness receive poorer care, have higher rates of misdiagnosis, and die up to 20 years earlier than those without mental illness, with stigma and fear among health professionals contributing to this inequality [1–4]. Stigma also shapes medical undergraduates’ career preferences, with some prematurely deciding that a psychiatric career is not for them, contributing to recruitment difficulties in mental health services [5, 6]. Attitudes to mental illness have personal implications for medical students, who are at higher risk of mental health problems themselves [7], yet avoid help-seeking, despite implications for their wellbeing and the care they provide to patients [8, 9].

Attitudes may be defined as evaluative thoughts, feelings, and behaviors towards an attitudinal object [10]. They can be hard to change, but are modifiable with repeated exposure to contradictory evidence [11]. Prior research is conflicting but indicates that attitudes towards mental illness may be malleable to change during the course of a psychiatric clerkship [6, 12–14]. Role models and early exposure to psychiatry may positively influence attitudes [15, 16]. However, existing research in this field is either cross-sectional, so does not monitor change, or does not evaluate why change arose. There have been calls for novel research designs to better understand how change in student perceptions may arise [17] with a view to informing curricula design. With this in mind, we used a longitudinal qualitative methodology to explore how students’ attitudes towards mental illness may evolve over time, and how their clerkship experiences may influence such changes.

## Methods

### Design

This longitudinal qualitative study explored medical students’ perceptions of mental illness through time. Conducted within an interpretivist paradigm, we sought to understand the diverse ways students made sense of their clerkship experiences and how this influenced their beliefs about mental illness, rather than assuming a single fixed reality. In line with this paradigm, an inductive approach was used, with qualitative trajectory analysis [18] to draw out convergence and divergence in students’ clerkship experiences and attitudes prior to, during, and following their clerkship.

Reflexivity was maintained throughout the analysis, with AM maintaining a field diary, and regular researcher meetings where biases were discussed to ensure transparency. AM, a psychiatrist, held both an insider (as a former medical student) and outsider (as a senior psychiatrist) perspective. This supported in-depth discussion but may deter students from making negative comments, and data interpretation could be influenced by personal experience. Therefore, to mitigate possible threats to validity, AM did not supervise or teach participating students and LB, a non-clinical social scientist, provided second coding, adding an additional perspective and ensuring analyses were authentic representations of the attitudes and beliefs of participants.

### Population and Sampling

Purposive sampling was used to recruit medical students who were completing a compulsory 6-week psychiatric clerkship within a government-funded hospital and/or community mental health team, in one of the seven localities affiliated with a university in the South West of the UK. All clerkships followed the same curriculum, and institutions adopted a standard clinical approach to the teaching of psychiatry.

All 106 students allocated to consecutive clerkships were emailed prior to their clerkship, with details of the study. Twenty-four (23%) registered their interest. Participants were purposively sampled from respondents, to maximize diversity according to gender, interest in pursuing a psychiatry career, the location of their clerkship, and whether they were schooled in the UK. Students’ travel expenses to interviews were reimbursed, but they were not otherwise renumerated. Ethical approval was obtained from the University of Bristol, Faculty of Health Sciences Research Ethics Committee.

The sample size was determined using information power [19]. Factors reducing the sample size requirement were the adoption of a focused study aim; the use of a relatively homogenous sample, given that all participants are medical students from a single program; and the use of thematic analysis. Weighed against this, the inductive approach, which allowed themes to emerge from students’ own experiences, rather than being constrained to a pre-existing framework, increased the sample size requirement. AM conducted the interviews and her experience of medical school (personal and professional) ensured in-depth, high-quality dialogue that offered rich data, enabling sufficient information power with a sample of 14.

### Data Collection

Each student was asked to complete three one-to-one interviews, once prior to, once during, and once after their clerkship. Interviews were conducted face-to-face on the university campus by AM, assisted by a topic guide, which was used flexibly. Open questions facilitated deep discussion of experiences, attitudes, and feelings of significance to the student, while meeting the study aims. The topic guide (available from the corresponding author on request) was adapted iteratively, based on prior interviews, to facilitate exploration of emerging themes. The pre-clerkship interview focused on initial perceptions of mental illness and expectations of the clerkship, the mid-clerkship interview examined real-time experiences and their relationship to attitudes, and the post-clerkship interview reflected on students’ overall experience and any attitudinal change. Interviews were audio-recorded, transcribed verbatim, and analyzed using N-Vivo 12.

### Analysis

Trajectory analysis was conducted as described by Grossoehme and Lipstein [18]. Each participant’s set of interview transcripts at each timepoint were collated, read, re-read, and coded together by AM, to allow individual change over time to emerge. Initial codes were labelled to capture students’ perceptions at each timepoint, experiences, and reflections. Initial codes were re-examined, looking for relationships between events and change (or lack thereof). These were then grouped into coherent themes, and tabulated according to theme and timepoint to evaluate change (or absence of change), and experiences related to that change in each individual. Transcripts from nine interviews (23%) were independently coded by LB, and themes were agreed between the researchers.

Themes were then compared across individuals, looking for convergence and divergence across experiences, and similar themes were grouped and renamed. The data were iteratively revisited to ensure that emerging interpretations remained grounded in the participants’ original responses. AM and LB met regularly during the analysis to discuss the themes emerging and both descriptive and interpretative analyses of the data. Further external perspectives and critical feedback were sought from PM and JS, helping to refine and validate the balance between descriptive accuracy and interpretive depth. The themes are presented as a series of discussion points.

## Results

Fifteen students completed baseline interviews, but one requested to withdraw from the study. Therefore, data from 14 students were analyzed. A total of 39 interviews were analyzed with midpoint interviews being completed for 12 students and end-point interviews for 13 students. Students for which interviews were not completed did not differ from the overall sample, by any recorded characteristic. Where interviews were not completed, this was due to practical constraints in organizing the interview at a convenient time for the student concerned.

During the 6-week clerkship, students received approximately 8 days of small group teaching (including seminars, case-based discussion, and role play) and 22 days of clinical exposure. Clerkship details are shown in Table 1. Students were predominantly female and schooled in the UK (see Table 1). All students successfully completed the clerkship.

**Table 1:** Characteristics of students included in the study at baseline, and their psychiatry placements

Student characteristics	
Gender	9 female 5 male
Location of schooling	11 UK 3 outside the UK
Openness to pursuing a career in psychiatry	8 open to the possibility of a psychiatry career 6 would not consider a psychiatry career
Placement characteristics	
Placement location	6 within the University City 8 outside of the University City
Main placement setting (all are government-funded NHS services)^a^	5 psychiatric inpatient ward 1 forensic psychiatry ward (low secure) 1 crisis team^b^ 1 consultation-liaison psychiatry team 6 split placement: psychiatric inpatient ward and either community^c^ (4), crisis (1) or consultation-liaison psychiatry (1) team

^a^Students had the opportunity to organize taster days outside their main placement setting (with child and adolescent, learning disability services or other services listed above). Students are predominantly hosted on inpatient wards and mixed community and ward placements; therefore, these proportions are reflective of the placements available

^b^Crisis teams work intensively with people in the community as an alternative to admission and to support early discharge

^c^Community teams provide long-term community support via outpatient clinics and home visits

Student accounts centered on four themes: stigma; patients as fragile; the emotional toll associated with encountering patients with mental illness; and the treatability of mental illness. Students recognized that their pre-existing beliefs had been influenced by the media, cultural norms, personal experience of illness (in themselves, friends, or family), and comments from friends and clinicians on previous clerkships, for example, “Before I wasn’t really sure what I was expecting. I think I was just going by what other people have said in passing about psychiatry… I had the kind of picture of One Flew Over the Cuckoo’s Nest and like Sarah Connor from Terminator 2.”

There was evidence of change in student beliefs between the first and second interviews, but little further change between the second and third interviews. Therefore, each theme is presented, comparing attitudes at baseline with those during follow-up interviews, at either timepoint. Anonymized quotations illustrating change in the attitudes of individual participants are presented in tables. Quotes illustrating the sources of such change are embedded within the text.

### Stigma

Stigmatized attitudes showed the greatest change over the course of the clerkship, initially increasing, then reducing with patient contact (see Table 2). Students expected patients to be different from themselves, from people with mental illness who they knew personally, and from other medical patients. They viewed patients as “violent” or “manipulative,” anticipating behavior which was “unpredictable” or “hard to understand.” These beliefs contributed to avoidance of the ward environment. Some students with a history of mental health problems believed that their personal experience had resulted in them holding less stigmatizing attitudes towards patients.

**Table 2:** Change in stigmatized attitudes regarding mental illness

Theme	Prior to clerkship	During/after clerkship
Patient contact reduced perceptions of patients as dangerous	“You know that you shouldn’t judge people and you know that there’s all this stigma around mental illness… but then, also, coming into contact with someone with a mental illness on the street… you don’t want to. I know it sounds awful, but… you do find yourself… just feeling a little bit scared.”	“The more that I’ve spoken to patients, the more I kind of understand that like, yeah, they can start to get a bit like, wound up or irritated—that doesn’t mean that they’re going to kick off and like, attack you… people are allowed to get irritated because they’re talking about difficult subjects, and you just have to sit and let them do that… you shouldn’t be scared of them.”
Patient contact increased empathy	“It is something so… very separate from everything else we’ve done… when I see someone on the streets… and they are going crazy you are just like ‘oh man, so bad’ and that is not… in the same way as if you saw someone limping, you’d be like ‘oh that looks hard’… It is more sort of ‘you are crazy’. It is distancing… like ‘I can’t engage with that’… It is hard to relate to so when you see someone else with a mental health problem… you kind of other them because it is hard to engage with”	“It’s a lot harder to distance yourself from patients than I expected it to be. Because whilst, you know, they’re saying crazy things… When you’re there you’re like… this is real for you”
Students believed personal experience of mental illness reduced stigma	“I feel more comfortable with psychiatry because I’ve seen some of the things and I’ve felt some of the things… and I feel like it’s more of… an issue of just getting them to talk to you… and just being a good listener.”	“I don’t see them [psychiatry patients] any differently to any other person… I think I understand mental health issues because I’ve been through it”
Students who lacked supervision described ongoing concerns about the dangerousness of patients	“It’s probably like the whole walking on eggshells. Until you really know what to expect… I don’t want to sort of have any pre-judgements… but at the same time a lot of what’s said about psychiatry isn’t necessarily that positive.”	“Doing medicine and surgery… you don’t really expect them to get up and chase you round the ward or smash a dinner plate like next to your head or something. Whereas… there’s a couple of patients that sort of have their refractory schizophrenia who are just angry.”
Hearing patients histories helped students empathize and view symptoms as illness	“The people can have quite manipulative traits… knowing how to cope with that… it’s a big unknown… I think it will be quite challenging”	“You find a lot more about a person and how they’ve developed… and the significant events in their life. A lot of them involve… abuse… I didn’t like judge them beforehand… [but] one of the defining features of this placement is that… It’s not… who they are as a person… they are ill.”

During the clerkship, differences in the clinical environment (for example, locked wards and the need for alarms) reinforced the belief that patients were different, unpredictable, and dangerous. Concerns were heightened when students were advised about safety measures to mitigate against possible physical risk from patients, e.g. wearing alarms.

However, over the course of the clerkship, students showed an improvement in their empathy and a reduction in stigmatized beliefs. Students who did not show stigmatized attitudes at baseline showed little attitudinal change. Despite similar beliefs at baseline, the students who had little patient contact showed a persistence of their stigmatized attitudes and concerns about engaging with patients throughout the clerkship.

#### Sources of Change

During the first week of the clerkship, student’s perception of people with mental illness as “different” was reinforced by aspects of the psychiatric setting which differed from medical and surgical wards, for example, “the environment’s so different… the ward was, like, double locked and it was pretty intimidating… it wasn’t like everyone was laying down in bed….” This created uncertainty about how to initiate consultations with patients. Concerns about patients being dangerous became heightened following advice about safety measures to mitigate against possible physical risk from patients, such as wearing alarms, for example, “They were like… ‘you don’t have to be scared, but… just in case you’re attacked, like here’s an alarm’… the first sort of few days I was a bit on edge.”

Students described how attitudinal change arose through clinical contact with patients, as described below. Students initially tried to fit clinical experiences with their existing stigmatized beliefs, but through repeated clinical encounters, their perceptions shifted.

“We were sitting near the door because that’s what they advised... he was talking about a lot of random things… he started approaching the door and I was like oh my god, this is what I was worrying about. But then... he was just like... ‘can you help me open the window?’ Like you know, any normal... person would do... I sort of realized that... I was being really discriminatory towards him by thinking that he was going to do something to me. Like that kind of made me realize… maybe I shouldn’t be so scared.”

Students explained that welcoming supervisors and other staff could allay students’ concerns and facilitate their contact with patients. Some students described difficulty accessing their supervisor, heightening uncertainty in the ward environment, and leaving them reluctant to see some patients, with little evidence of change in attitude over the clerkship. For example:

“You have an educational supervisor; I’ve barely seen him. I don’t know what I’m meant to be doing. It’s very easy to disappear like off the placement for a few days… [I’ve] clerked the… patients that are the easiest… to clerk.... [The] more violent ones… they’re both refractory schizophrenics… [I haven’t spoken to them because] you wouldn’t really get any sense out of him.”

Students attached to a single team described how being entrusted with clinical tasks gave them a sense of purpose, which encouraged patient contact, for example, “They make you feel like part of the team as opposed to kind of a spare part… [The team] need us to go see patients… It feels like you’re actually contributing, have things to sort of add.” Such students did not raise any concerns about a lack of variety, though taster sessions in other environments meant they still accessed varied experiences. Students who perceived themselves to have a supportive team and useful clinical role described being exposed to more clinical contact and reported experiencing greater satisfaction with their work, for example, “I cannot even describe how much a difference it makes when your supervisor knows where you are and the staff know where you are and expect you and give you jobs and you, you come out of the day like absolutely buzzing.”

Students reported that the academic stipulation for patient contact motivated them to see patients, though this could detract from students’ enthusiasm for clinical work, as described here: “I’m just doing it because I feel like I have to, to tick it off. It… takes the interest out of it, a little bit… [but] it’s good that portfolios do motivate you to get up in the morning… I just didn’t feel like, ready to [speak to patients]… but then actually once I did it, it was fine… it was just motivating myself to do it, which I found harder.”

Although teaching did not appear to influence attitudes directly, it sometimes helped students to build confidence. In particular, engagement with patients through roleplay and learning from people with lived experience proved to be particularly helpful. For example, “the patient kind of gave her little talk about what it’s been like and then we had the chance to like, ask her questions, which was really, really useful. Because it was kind of like, a safe environment to just ask someone about their experience.”

Students observed that among the staff, in the main, non-judgmental and empathetic attitudes to patients prevailed, and most of the students tried to emulate this. However, a minority of students witnessed negative staff attitudes towards some patients, particularly patients diagnosed with borderline personality disorder. This led to a persistence of strikingly negative attitudes towards this patient group.

“Everybody else… has a name apart from… the borderlines… they always refer to them as like the borderlines… In a way they’re almost labelled as like the pests?... There’s a few patients on the ward… [with] learning disabilities, but they’re learning behavior from the borderlines... See, I’m doing it now! You hear the nurses talking about actually… the borderlines are really bad news.”

### Patients as Emotionally Fragile

Prior to the clerkship, students believed patients would be more “emotionally fragile,” than those they have seen in other settings, heightening student apprehension about engaging with patients. This led many students to worry about the need to censor themselves. As the clerkship progressed, students described becoming more confident in their interactions with patients, and being less likely to perceive patients as fragile (see Table 3). Furthermore, students recognized the adversity which may have contributed to a patient’s poor mental health, and were also able to reflect on patients’ resilience. Yet, despite this, some students continued to worry about upsetting patients.

**Table 3:** Change in perceptions of people with mental illness as emotionally fragile

Theme	Prior to clerkship	During/after clerkship
Through patient contact, students recognized patients’ resilience	“[I’m] not looking forward to it, but I think it will be a challenge just because they’re going to have a completely different view in life, they’re going to have complete different responses and a lot of emotion”	“Once I actually did it, like, spoke to a few of the patients… I never felt like I was… tip toeing around them or having to… watch my words… They are the resilient people who fought through the different mental illness that they’ve had, fought through the abuse that they suffered, the environment that they’ve had, the neglect and, yeah. And that they’ve got to this point”
Students who had fewer interactions with patients expressed concerns throughout the placement	“[I’m] worrying about saying the wrong thing… I don’t want to upset anyone”	“You need to be a lot more careful with what you say… it just seems unfair that, to upset someone for kind of, me to get my clerking portfolio signed off… I just don’t want to make them feel worse than they already do”

#### Sources of Change

Students recognized the value of speaking to patients, and hearing their histories, and thought this had played an important role in changing their perceptions about patients, for example, “Patients have generally been really receptive. They don’t seem to mind… telling me lots [of] distressing… things… they seem to quite like talking.” Despite this, some continued to describe feeling worried and guilty when patients became upset in consultations, as described here: “[talking] kind of drags up a lot of things with people. So, it’s obviously hard to watch that and know that you kind of did that… I just feel guilty, yeah, or perhaps that you’ve done something wrong.”

Supervision, academic requirements, and face-to-face teaching all helped to prepare students and supported engagement with patients. Positive responses from patients also helped to build student confidence, for example, “Patients have said that… I’ve been very, very understanding and I’ve been compassionate, so I do feel like I’m doing a decent enough job.” Some students, who described a lack of supervision, and focus on meeting requirements with minimal patient contact, cited ongoing concerns about upsetting patients as a reason not to do more.

### Treatability of Patients

Before undertaking their clerkships, most students considered mental illness to be hard to treat and patients slow to improve (Table 4). However, some expressed hope change may be possible.

**Table 4:** Change in attitudes regarding the treatability of mental illness

Theme	Prior to clerkship	During/after clerkship
Beliefs that mental illness is hard to treat persisted	“What do you do with people who don’t get better?… if someone’s psychotic… You just put them away in a special center and I don’t know”	“I think I wouldn’t be able to find it as satisfying [as a career]… I don’t know how much of a difference you can make”
Students minimized the efficacy of treatments	“You want to fix things… ‘We did this to you… now you are going to be okay’… Whereas mental health is just much more of a longer process… you are dealing with things which are much broader… you are not making much headway”	“We can’t give them a drug to fix the fact that they’re abused… It’s a bit unfixable… You want to fix things… so you kind of medicalising it to try and be like oh, ‘I can provide this talking thing’… ‘I think you should have this medication’”
Some believed a holistic approach was valuable	“It’s such a stretched service, you don’t always have the time to go in and go… I’m just gonna take an hour and listen to you… it might not actually be something that’s actually fruitful, but… I, do want to… believe that it will help them feel a bit better”	“It’s something I am really considering as a potential career choice… In mental health… [it’s] important to understand the person rather than just the diagnosis… it’s more to do with helping the person however you can… they may not always get better but… when it does work [it’s] astonishing.”

During the follow-up interviews, students viewed the predisposing factors to illness as being beyond the control of the doctor, and thus concluded that treatment would be ineffective and that recovery was uncommon or slow to arise. Despite this, some believed that psychiatrists made a positive difference, praising the holistic nature of psychiatric care.

#### Sources of Change

Despite encountering patients who were recovering from episodes of illness, for many students, this experience did not impact their underlying belief that mental illness is hard to treat. Students expected patients to relapse. For example:

“There are patients where you see their progression with treatment… and then cycle just continues because we’re sending them back to the same environment that they came from... this inpatient stay is just… a plaster, it’s not a treatment… [However] it’s a bit biased representation because… we don’t usually get to see the patients who flourish at the end. Especially on a 6-week psychiatry block.”

These beliefs influenced attitudes towards psychiatry as a career choice. Even when contrasting psychiatry to other specialties where cure was unlikely, some students still perceived psychiatrists to have little impact on their patients’ lives, for example, “I always wanted to be in medicine… making a tangible difference in someone’s life… even if you’re not necessarily curing them, even palliative care… but how do you do that [in psychiatry]… if you know for a fact that you can’t make someone’s life better?”.

Others remained open to psychiatry as a career, viewing its more holistic approach as appealing. Students expressed mixed views about the feasibility of following patients up for longer, with some worrying that longer clerkships in multiple specialties running in parallel would make it difficult to fully immerse themselves in each clinical environment.

### Emotional Toll

Prior to the clerkship, students raised concerns that the distress associated with mental illness could exert a disproportionate emotional toll on clinicians (Table 5), though some thought they were unlikely to experience such distress themselves.

**Table 5:** Change in attitudes regarding the emotional toll of patients’ distress

Theme	Prior to clerkship	During/after clerkship
Concerns about emotional distress were allayed by positive experiences and a sense of purpose	“I’m more worried about seeing people upset and not being able to deal with it”	“I haven’t really been affected that much by people talking about things… I didn’t find it difficult because… I empathize, but at the same time it’s like you know that you’re there to… help them”
Some described choosing not to engage with a patient’s distress	“I’m quite good at separating myself and just saying, oh that’s really sad… and then… move on… If I don’t know the person, I find it very difficult to ask them to open up to me… I’ll take the easy way out and just do some book learning rather than actually getting experience on the wards.”	“His father had been physically abusive… it is difficult to hear, but… I’m completely separate from this patient, I’m not his doctor… [so] I don’t feel that [distress]… I couldn’t be bothered to… care about someone, because I was never going to see them again”
Some believed seeing multiple patients may exert an emotional toll	“It can be quite emotionally exhausting to go through that all day, and whether or not I’ll have any coping mechanisms left to myself, by the end of the day is also something to think about”	“It really puts your life in perspective and makes you feel better about the things you have. On the flip side, obviously it could be emotionally draining to talk to some of these patients all day, but I’ve enjoyed… listening to people’s stories.”
Some felt drained by the clerkship despite experiencing little distress with patients	“I’m not really a person who like brings it all home with me… [so] I don’t think it’s gonna be particularly traumatic. Like from what I’ve heard of my friends, like obviously some things are upsetting. But… I think it’ll be fine.”	“It’s a bit like emotionally draining… I’ve never been in a situation… that you think like, oh, this is really draining. But then you just notice at the end of the day… I just want to sleep… I think just cause it’s completely new”

During the clerkship, although students described experiencing empathetic feelings of sadness when hearing about patients’ lives, few reported experiencing distress lasting beyond the consultation. For some, this alleviated their concerns that patient contact had an emotional toll. Students who empathized with patients attributed the lack of lasting distress to feeling that they were there to help. Students who struggled to empathize attributed the absence of an emotional response to seeing themselves as different from the patient, and not having any clinical relationship with them.

Despite the absence of lasting distress among the students, some persisted in holding a belief that psychiatry is an emotionally exhausting career and two students described feeling “drained” by the clerkship.

#### Sources of Change

Students reported that being less active in the consultation could lead to them experiencing more distress. Some explained how, when leading consultations, they took on a professional persona and had less mental space to reflect on the patient’s story, distancing them from an emotional response, for example, “going and seeing patients… it hasn’t been difficult… cos I’m taking an active role in that… When you’re passively listening… it’s harder… I guess cos [when speaking to patients] I’m not dwelling, on their answer… cos I’m trying to formulate where I would take the conversation.”

This extended to non-clinical teaching experiences, e.g., seminars, which some students, with a personal experience of the mental illness, described as being more distressing than clinical work. Yet previous personal experience of mental health problems did not appear to influence distress experienced during consultations and such students felt it could aid understanding, for example, “[during consultations] you’re there in some sort of role and your responsibility at that time, I think, is to remain composed because, again, it’s not really about you… [but during role play] It’s like watching a film and I think everyone… internalizes things and, or thinks how is this relevant to me?.”

## Discussion

Understanding how students’ perceptions of mental illness evolve during the psychiatry clerkship is crucial for enhancing medical education, with significant implications for future patient care and the career choices of trainee doctors. Before beginning their clerkship, many students held stigmatized beliefs about mental illness, considered it untreatable and believed that psychiatric practice exerts a disproportionate emotional toll on doctors. Broadly, improvement in attitudes over the clerkship was facilitated by patient contact.

Although stigmatizing attitudes towards mentally ill patients reduced over the course of the clerkship, initial discussions about safety measures to mitigate possible physical risks from patients temporarily intensified these stigmatizing attitudes. This might explain some conflicting previous findings around the impact of clerkships on stigma [12–14]. We recommend risk be introduced elsewhere in the medical curriculum to reduce students’ perception that this is only relevant to psychiatry, as well as comprehensive preparatory discussions explaining the rationale behind safety interventions, and differences in the ward environment, to alleviate students’ concerns about working in psychiatric settings.

Improvements in the perceived dangerousness and fragility of patients were largely attributed to repeated, positive interactions with patients, emphasizing the critical importance of patient-student interaction. However, we could not determine the effect of witnessing aggression on student attitudes given it’s rarity. Early exposure to psychiatry has been associated with improvement in attitudes towards mental illness [20], and may exert its effect through building students’ confidence in psychiatric consultations earlier, thus encouraging further clinical contact. Curriculum designers seeking to reduce stigma should focus on normalizing engagement with mentally ill patients early in the medical school curriculum, to build students’ confidence in leading psychiatric consultations.

Students identified key facilitators to clinical contact, notably supervisors and welcoming clinical teams. Although the importance of supervisors offering feedback and encouraging reflection is recognized [21], their role prior to initial consultations was particularly valued by our participants. This finding aligns well with that from another recent study, which highlights how entrustment of students with clinical tasks is facilitated by supervisor time, experience, interest in teaching, and student-team relationships, while students’ fear and lack of preparation can pose barriers [22]. Students placed with a single team described feeling they had a clinical role. In contrast with surveys where students valued variety in clerkships [23], such students did not raise concerns about a lack of variety, though the option of arranging days in other specialties may have fulfilled this need. Single setting clerkships may also help identify and support disengaged students. Occasionally, clinical staff modelled stigmatized attitudes, reinforcing such beliefs in students, consistent with the literature on role-modelling [24].

Students valued teaching which closely mirrored clinical practice, such as role play. This echoes a systematic review in nursing students, which demonstrates the value of simulation to reduce anxiety, increase confidence, and prepare students for psychiatric clerkships [25]. Academic requirements could further motivate patient contact, and monitoring of goal achievement may also identify less engaged students. During clerkships, student-patient interactions are a priority, so supervisors and teams should be enabled with adequate time and support to engage with students and facilitate this.

The belief that psychiatric work imposes a disproportionate emotional burden on clinicians remains common, and may deter students from psychiatric careers, despite evidence suggesting higher levels of emotional exhaustion in other specialties [26]. Our study indicates that patient contact may have the potential to reduce concerns about the distressing nature of psychiatric practice where students feel they have helped patients. Notwithstanding, many students still harbored concerns about the emotional toll of psychiatric work, despite experiencing no lasting distress from clinical interactions. Supporting students to undertake meaningful roles in patient care and recognizing these contributions may facilitate attitudinal change, in addition to discussing the fulfilling nature of psychiatric careers as previously recommended [27]. Furthermore, students concerned about being “triggered” by patients’ distress may feel reassured by our finding that taking an active role in consultations may ameliorate their emotional impact, compared to being a passive observer.

The recognized perception of mental illness as being hard to treat [28] persisted through the clerkship, despite students sometimes observing patient improvement. In a 6-week clerkship, students may not have had sufficient time to witness recovery in their patient sample; however, previous quantitative studies suggest that change in perceptions of treatability cannot be explained by differences in clerkship duration alone [29–31]. In addition, the students were mainly exposed to acutely unwell hospitalized patients, requiring longer periods of time to fully recover [23]. The lack of attitudinal change in these circumstances aligns with the iterative reprocessing model which suggests attitudes change only with repeated exposure to contradictory evidence [11]. This could also explain the lack of broader attitudinal change among students who saw few patients. Highlighting where patients recover, enabling students to follow patients up after discharge, and involving recovered patients in teaching may help address this misconception. Although longitudinal integrated clerkships (longer clerkships with less frequent attendance in the specialty) may offer opportunities to witness recovery more frequently [32], students were concerned that such clerkships might impair their integration into a team, which could have other detrimental effects on attitudes. Further research could evaluate the effect of different clerkship models on attitudes.

The longitudinal design of this qualitative study allowed us to evaluate changing perceptions of mental illness and examine what evolving contextual factors students perceive to have influenced change across the psychiatry clerkship. This design reduced recall bias and identified curricular elements with potential to facilitate attitude change, which are of great relevance to educators. One-to-one interviews, and the building of trust and rapport across multiple interviews, facilitated students to openly share their views and personal experiences. However, social desirability may still have influenced student response and willingness to share negative views, particularly knowing the interviewer and main researcher was herself a psychiatrist. AM’s personal experience of clinical clerkships as a previous student and an educator may also have influenced the interpretations of the data, but understanding the structure and experience of psychiatry clerkships aided the collection of rich data during the interviews. Independent coding of a sample of interviews by co-author LB (a non-clinical senior researcher with a social science background) offered an additional perspective on the findings and ensured interpretations were true to students’ reports.

Our interpretivist approach sought to recognize the diversity of experience and attitudes among students, rather than attempting to unify the student experience. This approach recognizes students as individuals, and has allowed exploration of how change occurred through time. While our findings may be transferable to students with similar characteristics, qualitative research does not allow broad generalizations to be made, and the small sample size in this study limits the conclusions which can be drawn from our results. Purposive sampling was used to recruit participants with varied clerkship locations, degree of openness to a career in psychiatry, international status, and gender, increasing transferability. However, it should be noted that the study may have attracted students who held more favorable views towards psychiatry. A breadth of viewpoints were divulged, with good information power to meet the study aim. The 6-week clerkship represented most students’ first clinical experience of psychiatric settings, and the findings may be of less relevance to students with longitudinal clinical clerkships, as opposed to those that run as discrete blocks. Nonetheless, they may still reflect initial clinical experiences in longitudinal clerkships. Not all students spoke of their experiences of patients in other specialties; therefore, it is difficult to know the extent to which their attitudes are unique to patients with mental illness.

In conclusion, students’ stigmatized attitudes and concerns about the emotional toll of psychiatry improved during the course of the clerkship, but students continued to believe that mental illness is hard to treat. Students considered active engagement with patients to be most influential in helping to positively shape their attitudes towards mental illness. Furthermore, active engagement with patients exerted less emotional toll on students compared to simply being a passive observer. Students who felt supported engaged most effectively with patients, emphasizing the importance of supervision and engagement of all staff in facilitating student learning. Teaching which centered around clinical interactions built clinical confidence, while academic requirements further motivated clinical engagement. Attention should be given to demonstrating the effectiveness of treatment in facilitating patient recovery. Finally, given the importance of patient-student interactions in shaping attitudinal change, these should remain at the heart of psychiatric education.

## Data Availability

Anonymized quotes from the dataset supporting the conclusions of this article are included within the body of the article. The full transcripts generated and analyzed during the current study are not publicly available due to the risk this would pose to the anonymity of the individual participants. Excerpts of the transcripts relevant to the study are available from the corresponding author on reasonable request.

## References

[1] Public Health England. Health matters: reducing health inequalities in mental illness. 2018 https://www.gov.uk/government/publications/health-matters-reducing-health-inequalities-in-mental-illness/health-matters-reducing-health-inequalities-in-mental-illness. Accessed 20 June 2024.

[2] Chesney E, Goodwin GM, Fazel S. Risks of all-cause and suicide mortality in mental disorders: a meta-review. World Psychiatry. 2014;13:153–60. 24890068 10.1002/wps.20128 PMC4102288

[3] Hallyburton A, Allison-Jones L. Mental health bias in physical care: an integrative review of the literature. J Psychiatr Ment Health Nurs. 2023;30:649–62. 36740727 10.1111/jpm.12911

[4] Knaak S, Mantler E, Szeto A. Mental illness-related stigma in healthcare: barriers to access and care and evidence-based solutions. Healthc Manage Forum. 2017;30:111–6. 28929889 10.1177/0840470416679413 PMC5347358

[5] Fino E, Agostini A, Mazzetti M, Colonnello V, Caponera E, Russo PM. There is a limit to your openness: mental illness stigma mediates effects of individual traits on preference for psychiatry specialty. Front Psychiatry. 2019;10:755. 31736797 10.3389/fpsyt.2019.00775 PMC6833974

[6] Henderson C, Noblett J, Parke H, Clement S, Caffrey A, Gale-Grant O, et al. Mental health-related stigma in health care and mental health-care settings. Lancet Psychiatry. 2014;1:467–82. 26361202 10.1016/S2215-0366(14)00023-6

[7] Maser B, Danilewitz M, Guérin E, Findlay L, Frank E. Medical student psychological distress and mental illness relative to the general population: a Canadian cross-sectional survey. Acad Med. 2019;94:1781–91. 31436626 10.1097/ACM.0000000000002958

[8] Dyrbye LN, Eacker A, Durning SJ, Brazeau C, Moutier C, Massie FS, et al. The impact of stigma and personal experiences on the help-seeking behaviors of medical students with burnout. Acad Med. 2015;90:961–9. 25650824 10.1097/ACM.0000000000000655

[9] Tawfik DS, Scheid A, Profit J, Shanafelt T, Trockel M, Adair KC, et al. Evidence relating health care provider burnout and quality of care: a systematic review and meta-analysis. Ann Intern Med. 2019;171:555–67. 31590181 10.7326/M19-1152 PMC7138707

[10] Hitlin S, Pinkston K. Chapter 11: Values, attitudes, and ideologies: explicit and implicit constructs shaping perception and action. In: DeLamater JD, Ward A, editors. Handbook of social psychology. 2nd ed. New York: Springer; 2013. p. 319–39.

[11] Ehret PJ, Monroe BM, Read SJ. Modeling the dynamics of evaluation: a multilevel neural network implementation of the iterative reprocessing model. Pers Soc Psychol Rev. 2015;19:148–76. 25168638 10.1177/1088868314544221

[12] Fabiano N, Wong S, Miller A, Liu Z, Fiedorowicz JG. Changes in attitudes and confidence in the integration of psychiatry in other areas of medicine. Acad Psychiatry. 2022;46:593–8. 35701712 10.1007/s40596-022-01670-9

[13] Petkari E, Masedo Gutiérrez AI, Xavier M, Moreno KB. The influence of clerkship on students’ stigma towards mental illness: a meta-analysis. Med Educ. 2018;52:694–704. 29498433 10.1111/medu.13548

[14] Lyons Z, Janca A. Impact of a psychiatry clerkship on stigma, attitudes towards psychiatry, and psychiatry as a career choice. BMC Med Educ. 2015;15:34. 25888984 10.1186/s12909-015-0307-4 PMC4357197

[15] Kalindjian N, Hourantier C, Ludot M, Gilles de la Londe J, Corcos M, Cadwallader J-S, et al. Experiences of French medical students during their clerkship in adolescent psychiatry: a qualitative study. Eur Child Adolesc Psychiatry. 2023;32:1443–51. 35171376 10.1007/s00787-021-01940-1

[16] Archdall C, Atapattu T, Anderson E. Qualitative study of medical students’ experiences of a psychiatric attachment. Psychiatrist. 2013;37:21–4.

[17] Balon R. We should move past examining medical student attitudes and views of psychiatry. Acad Psychiatry. 2021;45:757–9. 34448121 10.1007/s40596-021-01526-8

[18] Grossoehme D, Lipstein E. Analyzing longitudinal qualitative data: the application of trajectory and recurrent cross-sectional approaches. BMC Res Notes. 2016;9:136. 26936266 10.1186/s13104-016-1954-1 PMC4776420

[19] Malterud K, Siersma VD, Guassora AD. Sample size in qualitative interview studies: guided by information power. Qual Health Res. 2016;26:1753–60. 26613970 10.1177/1049732315617444

[20] Holt C, Mirvis R, Bao J, Cross S, Hussain O, Hutchings H, et al. Three-year longitudinal follow-up of the Psychiatry Early Experience Program (PEEP): gaining and sustaining positive attitudes towards psychiatry in students at a UK medical school. Acad Psychiatry. 2019;43:600–4. 31372963 10.1007/s40596-019-01092-0

[21] Kilminster SM, Jolly BC. Effective supervision in clinical practice settings: a literature review. Med Educ. 2000;34:827–40. 11012933 10.1046/j.1365-2923.2000.00758.x

[22] Pinilla S, Kyrou A, Maissen N, Klöppel S, Strik W, Nissen C, et al. Entrustment decisions and the clinical team: a case study of early clinical students. Med Educ. 2021;55:365–75. 33301632 10.1111/medu.14432

[23] Halder N, Mulliez Z. Encouraging recruitment into psychiatry: practical initiatives. BJPsych Bull. 2021;45:15–22. 32513344 10.1192/bjb.2020.53 PMC8058864

[24] Wear D, Skillicorn J. Hidden in plain sight: the formal, informal, and hidden curricula of a psychiatry clerkship. Acad Med. 2009;84:451–8. 19318777 10.1097/ACM.0b013e31819a80b7

[25] Øgård-Repål A, De Presno ÅK, Fossum M. Simulation with standardized patients to prepare undergraduate nursing students for mental health clinical practice: an integrative literature review. Nurse Educ Today. 2018;66:149–57. 29704702 10.1016/j.nedt.2018.04.018

[26] Prentice S, Dorstyn D, Benson J, Elliott T. Burnout levels and patterns in postgraduate medical trainees: a systematic review and meta-analysis. Acad Med. 2020;95:1444–54. 32271234 10.1097/ACM.0000000000003379

[27] Brenner AM, Balon R, Coverdale JH, Beresin EV, Guerrero APS, Louie AK, et al. Psychiatry workforce and psychiatry recruitment: two intertwined challenges. Acad Psychiatry. 2017;41:202–6. 28205068 10.1007/s40596-017-0679-3

[28] Mukherjee R, Fialho A, Wijetunge A, Checinski K, Surgenor T. The stigmatisation of psychiatric illness: the attitudes of medical students and doctors in a London teaching hospital. Psychiatr Bull. 2002;26:178–81.

[29] Bulbena A, Pailhez G, Coll J, Balon R. Changes in the attitudes towards psychiatry among Spanish medical students during training in psychiatry. Eur J Psychiatry. 2005;19:79–87.

[30] Xavier M, Almeida JC. Impact of clerkship in the attitudes toward psychiatry among Portuguese medical students. BMC Med Educ. 2010;10:1–8. 20678213 10.1186/1472-6920-10-56 PMC2919544

[31] Lyons Z. Impact of the psychiatry clerkship on medical student attitudes towards psychiatry and to psychiatry as a career. Acad Psychiatry. 2014;38:35–42. 24464416 10.1007/s40596-013-0017-3

[32] Cheng E, Hirsh D, Gaufberg E, Griswold T, Wesley BJ. Findings from the Harvard Medical School Cambridge Integrated Clerkship, a year-long longitudinal psychiatry experience. Acad Psychiatry. 2018;42:357–61. 28646485 10.1007/s40596-017-0742-0

